# Survive and Thrive in Brazil: The Boa Vista Early Childhood Program: study protocol of a stepped-wedge, randomized controlled trial

**DOI:** 10.1186/s13063-020-4217-3

**Published:** 2020-05-07

**Authors:** Alexandra Brentani, Ana Paula Scolezze Ferrer, Luana Bessa, Susan Chang, Susan Walker, Christine Powell, Jena Hamadani, Sandra Grisi, Günther Fink

**Affiliations:** 1 Department of Pediatrics, University of São Paulo Medical School, Av. Dr. Emeas de Carvalho Aguiar, 467, São Paulo, Brazil; 2 Caribbean Institute of Health Research, University of the West Indies, Kingston, 7 WI Jamaica; 3 International Centre for Diarrhoeal Disease Research, Bangladesh, 68 Shaheed Tajuddin Ahmed Sarani Mohakhali, Dhaka, 1212 Bangladesh; 4 Department of Epidemiology and Public Health, Swiss TPH and University of Basel, Socintrasse 57, 4002 Basel, Switzerland

**Keywords:** Child development, Parenting interventions, Infant mortality, Neonatal mortality, Early childhood development

## Abstract

**Background:**

A growing body of evidence suggests that early life health and developmental outcomes can be improved through parental support programs. The objective of this project was to test the feasibility, impact, and relative cost-effectiveness of an adapted “Reach Up and Learn” program delivered through home-visiting programs as well as through center-based parenting groups on child health and development in the municipality of Boa Vista, Brazil.

**Methods:**

A randomized, stepped-wedge design was used to roll out and evaluate the two parenting platforms in Boa Vista municipality. A total of 39 neighborhoods with a high Neighborhood Vulnerability Index were selected for the study. For the first phase of the program, nine neighborhoods were randomly selected for home visits, and two were randomly selected for the center-based parenting groups. In the second phase of the program, 10 neighborhoods were added to the home-visiting program, and eight were added to the center-based program. In the final phase of the program, the remaining 10 control areas will also be assigned to treatment. Study eligibility will be assessed through a baseline survey completed by all pregnant women in the 39 study areas. Pregnant women will be eligible to participate in the study if they are either classified as poor, were under age 20 years when they became pregnant, or if they indicate to have been exposed to domestic or sexual violence. To assess program impact, an endline survey will be conducted when children reach age 2 years. The primary study outcome is child development at age 2 years as measured by the PRIDI instrument. Secondary outcome will be infant mortality, which will be assessed linking municipal vital registration systems to the program rollout.

**Discussion:**

This trial will assess the feasibility and impact of parenting programs rolled out at medium scale. The results from the trial should create evidence urgently needed for guiding Brazil’s national Criança Feliz program as well as similar efforts in other countries.

**Trial registration:**

ClinicalTrials.gov, ID: NCT03386747. Registered on 13 December 2017. All items of the World Health Organization Trial Registration Data Set are available in this record.

## Background

Brazil has made remarkable progress with respect to child nutrition and child survival over the past decade, with particularly impressive results in large urban areas. Infant mortality has dropped by roughly 50% since 2000 to 14 deaths per 1000 births across the country [1]. Most of the remaining mortality occurs during the neonatal period. More than 25,000 newborns are estimated to die each year within the first 28 days of their life in Brazil, with most deaths occurring in the first 7 days after birth. Previous studies suggest that one of the most effective ways to prevent such deaths is home-visiting programs, which support mothers in the first weeks of their infant’s lives, promote breastfeeding and kangaroo-mother care, and ensure appropriate medical care when needed [2–6]. From a child health and development perspective, the best outcomes have generally been achieved when continued support was provided to mothers from pregnancy throughout the first years of children’s lives. A nurturing and stimulating environment sensitive to maternal needs as well as the child’s health, nutritional and emotional needs is not only essential for healthy development but can also mitigate harmful effects of risk factors related to poverty and lack of parental resources [7–17].

Parental programs to promote nurturing environments have been successfully implemented in a range of low- and middle-income countries, including UNICEF’s *Care for Child Development (CCD)* framework, Jamaica’s *Reach Up and Learn* program, *Early Head Start* in the USA and the UK’s *Sure Start* [9, 18–22].

Brazil has successfully implemented its “family health strategy” – a health-focused home-visiting program coordinated by primary care units – since 2006 [23–25]. In 2016, the government passed the “Criança Feliz” law to further increase the support provided to vulnerable families [26]. Even though the program foresees home visits to all vulnerable families, there is very little guidance on how such programs should be implemented in Brazil, and what kind of content should be promoted.

In this project, we assess the scalability of a locally adapted version of an early childhood program previously developed in Jamaica. Between 2014 and 2016, the Jamaican “Reach Up and Learn” curriculum was adapted to the Brazilian context and tested through a small randomized trial in São Paulo (ClinicalTrials.gov, ID: NCT0270400). The content, delivered through fortnightly home visits, comprises parenting-skill tips as well as child stimulation activities supported with basic toys, addressing motor, social emotional, and cognitive development and language for families with children aged from 9 to 36 months.

While the reach of this program was not universal in this original trial, the estimated impact on participating mothers was large, and the program was well received at the local level (manuscript with main results under review). While this curriculum was delivered through home visits in the original study in São Paulo, recent evidence from Bangladesh [6] suggests that similarly positive impacts can be achieved through center-based parenting groups that are substantially cheaper from a logistical perspective.

**Fig. 1:**
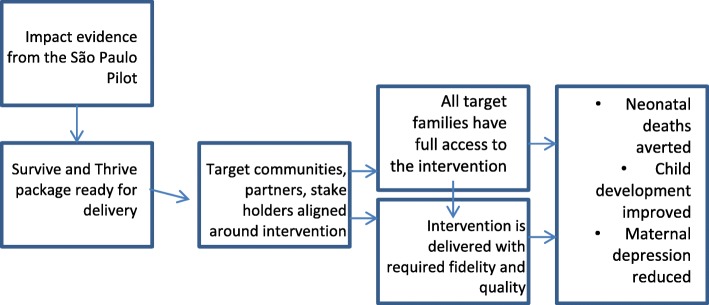
Theory of change

Given this and given also that the municipality had previously invested in a center-based model, it was decided to test both delivery platforms within this project. Given the relatively high burden of neonatal mortality in the study area, we also decided to start the intervention earlier. By enrolling pregnant women at the beginning of their third trimester, the revised intervention program aims to improve support for mothers during the antenatal and neonatal periods with the ambition to increase health service utilization and reduce the risk of adverse birth outcomes. Figure 1 presents the theory of change through which the curriculum may improve child survival and development.

### Objectives

The objective of this study is to rigorously assess the impact of an extended version of the previously tested and validated parenting curriculum on child survival and development in Boa Vista municipality. Through a three-arm trial, we will assess home visits through child development agents (intervention 1) as well as center-based parenting groups (intervention 2) relative to a randomly selected control group. Through a stepped-wedge rollout approximately one third of communities will receive the intervention in phase 1 of the rollout; in phase 2 of the program, two thirds of communities will be selected for interventions. All areas will receive interventions in the last phase of the program.

The specific study objectives are:

1. To evaluate the effect of home- and center-based parenting programs on child survival and development
2. To assess the impact of both programs on parenting behavior and practices
3. To evaluate the implementation process
4. To estimate program cost and cost-effectiveness
5. To assess relative program, reach among extremely poor and immigrant populations
6. To assess program spillover on older siblings in the household

### Trial design

The study was designed as a stepped-wedge, cluster randomized controlled trial with three arms. Arm 1 represents intervention delivered through fortnightly home visits; Arm 2 consists of the same content delivered through fortnightly center-based group meetings; Arm 3 is the control group.

To avoid contamination, neighborhoods were used as units of randomization. All 53 neighborhoods in Boa Vista were classified according to the municipality vulnerability criteria. Only neighborhoods classified as B (medium vulnerability) and C (high vulnerability) will be targeted by the program. As of 2017, 42 neighborhoods were in this category.

**Fig. 2:**
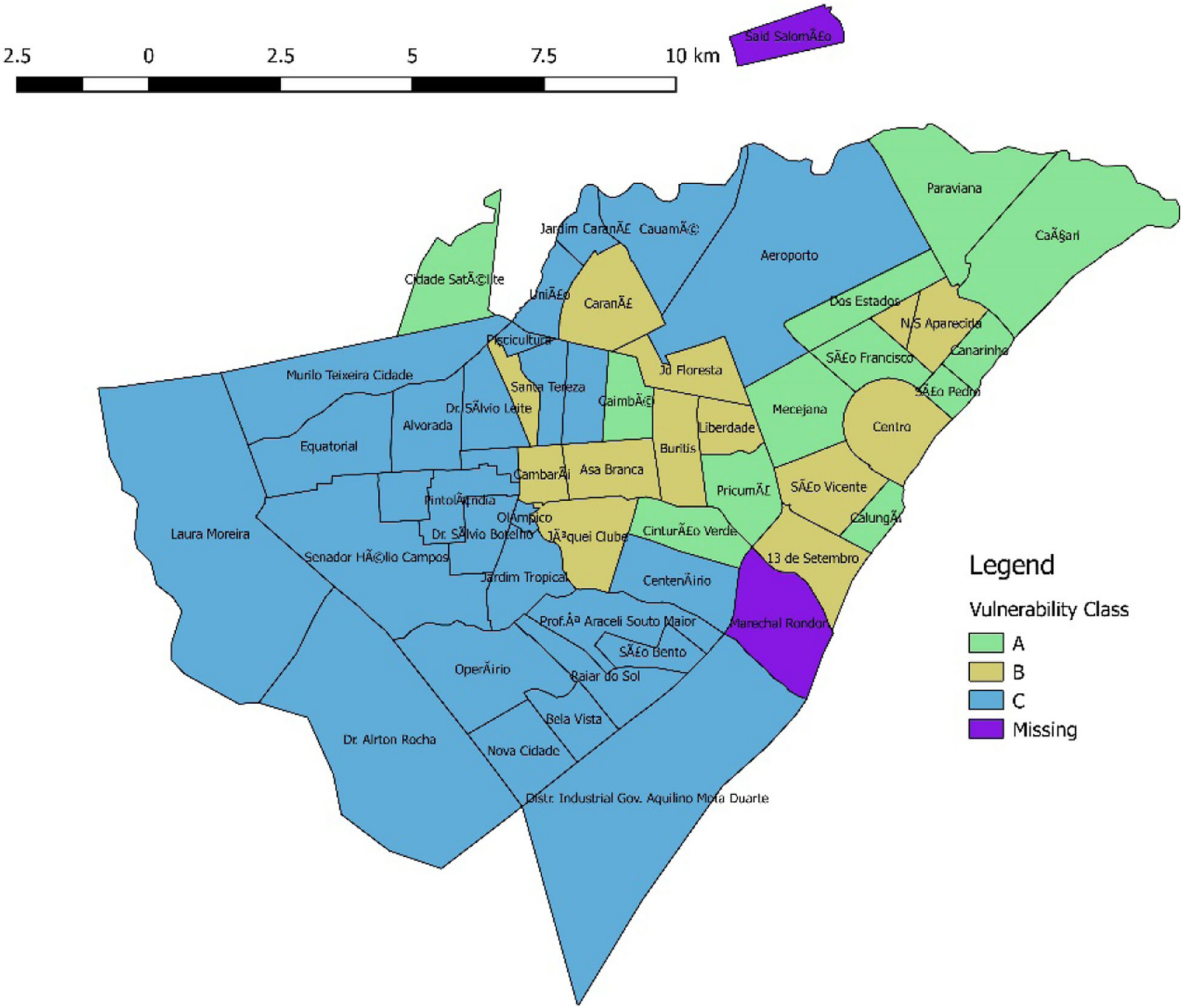
Neighborhood Vulnerability Index

Three neighborhoods had either existing home-visiting programs or other planned interventions, and were excluded from the study, leaving a final study sample of 39 neighborhoods. Figure 2 presents the neighborhoods vulnerability distribution.

From a programmatic perspective, the *Survive and Thrive in Brazil – The Boa Vista Early Childhood Program* eventually aims at providing support to all children born in the municipality of Boa Vista from the end of the second trimester of pregnancy until the children reach age 3 years. This program was designed to closely align with the national “Criança Feliz” program (Legal Framework of Early Childhood, Law 13.257/2016) [23], which will also provide partial funding for this initiative.

## Methods

### Study setting

The city of Boa Vista has a population of approximately 330,000 people, with, 22,377 families (~ 30%) currently receiving or enrolled in social cash transfers under the Bolsa Familia Program, very similar to the national average (35% of the population) [27]. According to the municipality health secretariat, infant mortality rate is estimated at 14.2 per 1000 live births, with 21% of births by teenage mothers and 11% of infants born preterm [28].

### Eligibility criteria

Study eligibility is restricted both spatially and at the individual level. As mentioned in the “Study design” section, the scale-up targets only vulnerable neighborhoods. Boa Vista social services classify all neighborhoods based on literacy rates, average per capita income (less than one quarter of the minimum wage), access to water, sanitation, sewage systems, and electricity into A (low vulnerability), B (medium vulnerability), and C (high vulnerability) areas. Out of the 55 neighborhoods in Boa Vista, 12 were classified as “high socioeconomic status” (A), 14 were classified as “low vulnerability” (B), and 29 were classified as “high vulnerability” (C). The study was restricted to the 43 B and C areas^1^.

Within all target areas, all pregnant women as well as women with children under the age of 1 year are eligible for the program as long as they are either poor, were under the age of 20 years at the time of conception or were ever exposed to domestic or sexual violence.

### Interventions

#### Home visit arm (Arm 1)

The intervention will be delivered during fortnightly home visits with the presence of the child and at least one of the main caregivers (the program focuses on the mother, but ideally with paternal participation or any other family member who routinely spends time with the child). The home-visiting curriculum is divided into three main modules: (1) the pregnancy module: this module is designed to make mothers aware of pregnancy danger signs, to encourage adherence to antenatal care (attendance, performing exams, and supplementation), to improve bonding and positive parental practices and to prepare women for breastfeeding; (2) the neonatal module: comprises three home visits during the baby’s first month of life. One visit during the first week, a second visit at 15 days and the third visit at 28 days. The primary focus of these visits is babies’ health, breastfeeding and bonding; and (3) the child module for ages 2–36 months. Each home visit has three or four play activities to address child development domains (gross motor, fine motor, language, cognitive development, and social-emotional development). A recycled-materials toy kit is used to support these activities. Visits take in average 45 min; after demonstrating and practicing each activity with the caregiver, the visitor leaves the toys, and mother/caregiver is encouraged to play and interact with the child in the 2-week interval between the visits. Activities are age-appropriate, and toys and learning materials are exchanged for a new set at each visit. Home visits will be conducted by newly hired and trained child development agents, who will be tasked to complete 60 home visits per month (three visits per workday), supporting 30 families.

#### Group-meeting arm (Arm 2)

Group meetings are designed to deliver essentially the same content. Meetings are held fortnightly at the CRAS center (Social Services Centers) for groups of eight mothers and their children, other members of the family can participate as well. Groups are formed with participants at a similar stage of gestation or with children of similar age. Group composition remains fixed to increase bonding over time. The only difference between the center and the home-based curricula is the number of activities per session: due to the larger number of participants, the number of activities is reduced to two at the centers. The neonatal module is also reduced to one or two sessions since we do not expect mothers to attend the sessions during the first 2 weeks of the babies’ lives. Group moderators will be hired by the project and will be tasked to schedule three age-group-specific 1-h and 30-min sessions each day.

Program teams will be based at the seven regional social service units (CRAS) and will deliver the intervention according to the services’ coverage area. Group meetings will be held at the centers, where a meeting room will be fully dedicated to the project for ease of access to mothers since the rooms are distributed within the more vulnerable areas.

Attendance at the sessions, dropouts and refusals will be registered in the intervention logs and monitored by the team on a monthly basis. In order to improve adherence, if a participant skips three consecutive sessions, she will be contacted by the delivery team supervisor to understand the reasons and eventual problems that need to be addressed to continue participation. The dropout log registers the participants’ motives for discontinuity and, depending on these, the supervisor can also contact the participant to revert dropout.

#### Control group (Arm 3)

Pregnant women living in the control-group-allocated neighborhoods will receive the regular public services provided by the municipality. In terms of health care, they will receive primary, secondary and tertiary care through the primary care units and hospitals run by the municipality or state secretariat. They will also have access to the regular social services provided by the Social Services Units and to other social (and cash transfer) programs such as the Bolsa Família.

### Modification of interventions or protocols

After the trials have started, there will be no special criteria for discontinuing or modifying the allocated interventions. The research group will monitor, on a monthly basis, protocol-adherence aspects such as group allocation, participation, logs, and other registry instruments and will participate in the weekly delivery team meetings and supervise home visits and group meetings.

No protocol amendments are expected during the trial but the private investigator (PI) will notify funders, the municipality and the Institutional Review Board (IRB) if any changes should be needed. Any deviations from the protocol will be documented using a breach report form and the protocol will be updated in the clinical trial registry.

### Adherence

Program rollout will be closely monitored by the Steering Committee. Before the trial launch, the municipality agreed not to deliver similar concomitant interventions for this population in the selected neighborhoods. To ensure that this will not happen in practice, the research group will collect information on other social program participation.

### Concomitant and post-trial care

Implementing the strategies of the ECD intervention (groups meeting and home-visiting) will not require alteration to usual care pathways (including use of any medication). Standard medical and child support care will be available to all study arms throughout the trial. There is no anticipated harm and compensation for trial participation during or after the trial. All participants will continue to have regular access to public health and social services once the trial is over. The continuity of the intervention after the trial is dependent on mayoral discretion.

### Outcomes

The primary study outcome is child development at age 2 years as assessed by the PRIDI Scale. The secondary study outcome is child survival, which will be assessed using data from the municipality’s vital registration system (SIM and SINASC). Maternal depression will be assessed using the Center for Epidemiologic Studies Depression Scale (CES-D Scale).

Intention-to-treat analysis will be used to compare outcomes across the three study arms.

Additional per-protocol analysis will be conducted using data on program compliance. Our primary measure of compliance in the home-visiting arm will be the number of home visits completed. Our primary compliance measure in the center-based groups will be the number of sessions attended by the mother. Table 1 presents the data collection overview.

**Table 1:** Data collection overview

Data collection phase	Instruments
Baseline (12,000)	Participant identification, anthropometrics, and habits Socioeconomic characteristics Household characterisits Previous gestational information Maternal depression (EPDS) Neighborhood characteristics and social support Couples’ conflict Parental knowledge Maternal cognitive development (WAIS) Paternal characteristics
Main endline (*N* = 3000)	Child development (PRIDI) – Primary outcome Maternal depression (CES-D) Caregiver self efficacy (PSOC) Parental style (ACT) Parental knowledge Child anthropometrics (height and weight)
In-depth endline assessment (*N* = 240)	Attachment and caregiver interactions (OMCI) Bayley Scales for Infant and Toddler Development (BSID-III)
Qualitative interviews	Focus groups with child development agents and group-meeting facilitators Individual interviews with program beneficiaries divided by full compliers, non-compliers, and dropouts by intervention study arm

*ACT*, *BSID-III* Bayley’s Scales for Infant and Toddler Development, *CES-D* Center for Epidemiologic Studies Depression Scale, *EPDS* Edinburgh Postnatal Depression Scale, *OMCI* Observation of Mother-child interaction, *PRIDI* Regional Project on Child Development Indicators, *PSOC* Parenting Sense of Competence Scale, *WAIS* Weschsler Adult Intelligence Scale

### Randomization

**Fig. 3:**
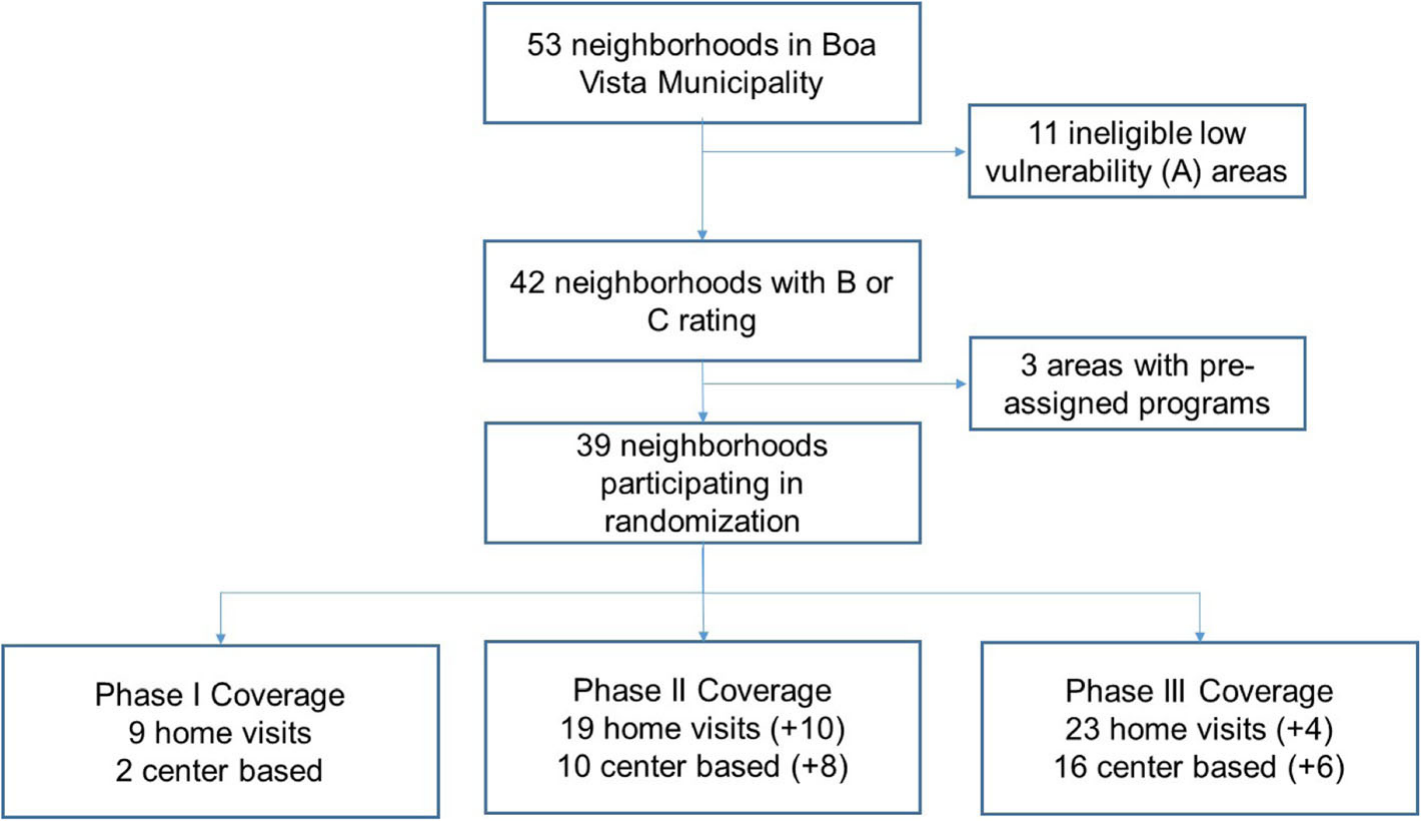
Neighborhood selection and randomization

**Fig. 4:**
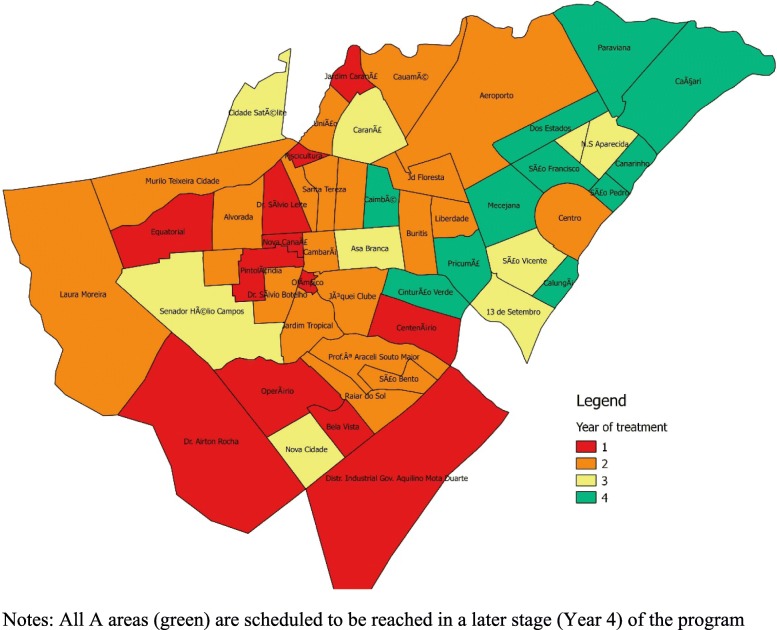
Spatial distribution of program rollout phases. Notes: all A areas (green) are scheduled to be reached in a later stage (year 4) of the program

Randomization of program rollout timing was based on a simple random-number draw in Stata. The number of treated areas in phases 1 and 2 was determined based on logistical feasibility and an initial agreement between the municipality and the project team: in phase 1, nine areas were chosen for home visits and two for center-based programs. In phases 2 and 3, program reach was gradually increased to reach all communities as outlined in the “Study design” section above. In phase 1, 11 neighborhoods will receive the intervention; in phase 2, 27; in phase 3, all B and C neighborhoods will receive the interventions. Figure 3 summarizes the neighborhood selection process and shows the random allocation of interventions by phase. Figure 4 shows the spatial location of areas chosen for each intervention phase.

### Recruitment

Recruitment for this project will be implemented in close collaboration with the Municipality Secretariat for Social Wellbeing and Health (SSWH). The SSWH identifies eligible pregnant women each month, and links them to the project team. Each woman is then visited by study staff and, conditional on their consent, completes a baseline interview. According to the municipality of Boa Vista, there are approximately 600 new pregnancies per month. The baseline forms are then used to establish eligibility for the program. As stated above, the following three (non-mutually exclusive) groups of women are eligible for the program:

1. Household classified as poor
2. Pregnant women under age 20 years (teenage pregnancies)
3. Women with previous exposure to partner violence

In the first few years of the program, a small sample of women with children under age 1 year will also be enrolled. This sample will be used to assess the relative impact of reduced exposure to the interventions.

In order to achieve enrollment to reach the sample size, the research group hired and trained a team of interviewers that will visit the eligible participants from the municipality list, but also actively search for other potential participants in the field. The research team will use the municipality official birth records on a monthly basis to monitor any unenrolled new children born in the catchment areas and enroll them afterwards.

Given that we will have 12,000 participating households in the intervention, we do not foresee any difficulties reaching the 3000 sample-size target for the endline.

#### Blinding

Given the nature of the intervention, blinding of participants is not possible. Endline interviewers will be blind to group assignment.

### Monitoring and potential harm

Given that the intervention does not present a risk for the population and that the municipality has committed to delivering interventions following the established timeline, there are no formal stopping rules for this trial. We also do not foresee needing any interim analysis for such purposes. However, even if not related to the intervention, the home visitor or group facilitator reports, or is informed of, any adverse event such as domestic violence, or health risk, which will be reported to the supervisor and to the Social Service Unit manager and relevant regulatory bodies as required, indicating expectedness, seriousness, severity, and causality.

### Sample size and power calculations

Based on the estimated number of births in the targeted B and C areas, we anticipate completing approximately 12,000 baselines (screening interviews) over the project period. Out of these mother-child dyads, we will randomly select 3000 for the endline assessment. We assume an average causal effect of 0.5 standard deviations (SD) for compliant mothers. With an anticipated average compliance rate of 50%, the study is powered to detect an intention-to-treat effect of 0.25 SD with power 0.9 between each of the two intervention arms and the control arm, assuming an average sample of 120 households in each of the 11 neighborhoods treated in phase 1, and an average sample of 60 households in each of the 28 control neighborhoods, assuming an intra-class correlation of 0.02.

In order to assess spillovers within households, we will also assess all older siblings under age 4 years in households selected for endline. Based on the high fertility rates observed at baseline, we anticipate a sample of 750 older siblings. Assuming a uniform distribution of siblings across clusters, the study is powered to detect a 0.3-SD change in PRIDI score with power 0.8.

In order to assess domain-specific changes in children’s development, we will also invite 80 mothers from each arm for a detailed assessment using Bayley’s Scales for Infant and Toddler Development (BSID-III). For these assessments, we will randomly select mothers from the control group as well as compliant mothers from the two intervention arms.

### Timeline

Baseline survey collection started on 1 December 2017 and will be finished by July 2020. The home-visits team was trained in November 2017 and visits were launched shortly after initiation of baseline in the phase-1 home-visiting areas; the first births within this group occurred in February 2018. Due to logistical challenges, center-based programs were started later; the two areas randomly selected for phase 1 of the project were formally launched in July 2018. Phase 2 (both for home visits and center-based programs) was started in July 2019, with 10 neighborhoods added to the home-visiting programs, and eight added to the center-based programs.

Endline surveys are scheduled to start in April 2020 and will target children at age 2 years. With the targeted sample size of 3000 households we anticipate to complete endline by July 2021. BSID-III assessments will start in July 2020 and should be completed within 6 months.

### Data management

Baseline and endline data will be collected in electronic format on tablets and stored on a secure server. The project leader will be responsible for the anonymization of the data system. A unique identifier will be used in the data system to preserve participant privacy and confidentiality.

### Statistical methods

For statistical analysis, standard regression models will be used for the continuous PRIDI outcome. Logistic regression models will be used for the secondary outcome of child mortality. All analysis will be conducted using the Stata 15 statistical software package. Primary analysis will be intention-to-treat. To assess the impact of partial compliance, we will also estimate average treatment effects on the treated (per-protocol analysis).

### Ethical clearance

The study was approved by the researcher’s IRB under protocol number CAAE:73722917.4.0000.0076 in August 2017.

### Consenting

Each participant signs the consent form at enrollment, during the baseline interview visit. Should the participant be a minor, the consent form is signed by a legal representative. On the consent form, participants will be asked if they agree to use of their data should they choose to withdraw from the trial. Participants will also be asked for permission for the research team to share relevant data with people from the universities taking part in the research or from regulatory authorities, where relevant. This trial does not involve collecting biological specimens for storage.

The consent form is signed in two copies so that the participants can keep a copy of the signed consent form. The PI’s, the social services’ responsible and the IRB’s contact information are provided in the consent form so that participants can find these in case of questions or other issues. Copies of the consent form are available from the corresponding author on request.

## Trial status

As of November 2019, trial enrollment is open and is scheduled to continue until July 2020. The present protocol is in its first version and has been since November 2017.

## Discussion

A large body of evidence has highlighted the importance of early parenting and nurturing care on child development. However, equitable early childhood programs and public policies are crucial for ensuring continued progress in Brazil’s efforts as well as other international efforts [11, 29–31]. In 2016, our group adapted and evaluated a home-based early childhood stimulation program with positive impact on child development. The proposed project aims to build on that evidence, creating a program that addresses not only child development, but child survival as well. We will evaluate the feasibility, impact, and cost-effectiveness of this program at the municipality level. The proposed project will tackle the two most salient problems for children age under 5 years in Brazil: the continued high rates of neonatal mortality, and the large disparities in early childhood development. Children growing up in poor urban areas of Brazil continue to be exposed to a substantial amount of adversity in early childhood due to exposure to pollutants, external and domestic violence, unstable family environments, maternal depression, and inadequate learning opportunities. Early disadvantages appear to be particularly great in illegal slum settlements characterized by poor hygiene, high levels of environmental pollution, high levels of community violence and, in many cases, also social isolation, often resulting in a high incidence and prevalence of maternal depression. Our aim is to use this transition-to-scale project to provide information to the Brazilian Government, contributing to these new national efforts. If successful, the tested interventions can potentially be used at the regional level and also nationwide and can ideally help Brazil to further accelerate its positive trends in neonatal survival and to help create a healthy, early life environment for all children in the country.

## Data Availability

The data sets generated and/or analyzed during the current study will not be publicly available until the end of the project but are available from the corresponding author on reasonable request.

## Footnotes

1 Three of these areas were chosen for municipality programs and excluded from the main study.
